# Spatio-temporal analysis of malaria incidence and its risk factors in North Namibia

**DOI:** 10.1186/s12936-023-04577-4

**Published:** 2023-05-06

**Authors:** Remember Ndahalashili Katale, Dibaba Bayisa Gemechu

**Affiliations:** Department of Mathematics, Statistics, and Actuarial Science, Faculty of Health, Natural Resources and Applied Sciences, School of Natural and Applied Sciences, Namibia University of Science and Technology, Windhoek, Namibia

**Keywords:** Spatiotemporal, Heterogeneity, Hierarchical Bayesian CAR model, Posterior mean, Malaria incidence

## Abstract

**Background:**

Millions of dollars have been spent in fighting malaria in Namibia. However, malaria remains a major public health concern in Namibia, mostly in Kavango West and East, Ohangwena and Zambezi region. The primary goal of this study was to fit a spatio-temporal model that profiles spatial variation in malaria risk areas and investigate possible associations between disease risk and environmental factors at the constituency level in highly risk northern regions of Namibia.

**Methods:**

Malaria data, climatic data, and population data were merged and Global spatial autocorrelation statistics (Moran’s I) was used to detect the spatial autocorrelation of malaria cases while malaria occurrence clusters were identified using local Moran statistics. A hierarchical Bayesian CAR model (Besag, York and Mollie’s model “BYM”) known to be the best model for modelling the spatial and temporal effects was then fitted to examine climatic factors that might explain spatial/temporal variation of malaria infection in Namibia.

**Results:**

Average rainfall received on an annual basis and maximum temperature were found to have a significant spatial and temporal variation on malaria infection. Every mm increase in annual rainfall in a specific constituency in each year increases annual mean malaria cases by 0.6%, same to average maximum temperature. The posterior means of the time main effect (year t) showed a visible slightly increase in global trend from 2018 to 2020.

**Conclusion:**

The study discovered that the spatial temporal model with both random and fixed effects best fit the model, which demonstrated a strong spatial and temporal heterogeneity distribution of malaria cases (spatial pattern) with high risk in most of the Kavango West and East outskirt constituencies, posterior relative risk (RR: 1.57 to 1.78).

## Background

Malaria transmission remains unstable in most of the high and moderate endemic malaria nations where climatic factors are known to be mostly associated with malaria incidence from temporal and spatial perceptive [1]. This disease kills over 750,000 people each year in African and Asian countries, mostly children and pregnant women, with 435,000 deaths among children under the age of five, particularly in most of the African countries including Namibia [2–5]. Namibia is one of the countries aiming to meet the third Sustainable Development Goal (SDG 3), which calls for the end/elimination of the malaria epidemic by 2030 [6–9]. The Namibia Malaria Indicator Survey (MIS) along with a similar funded study were carried out in 2009. National malaria strategy was also implemented from 2010 to 2016 with the goal of reducing malaria cases from 13 to less than 1 cases per 1000 population by 2016 and be malaria free by 2020 [10–12], all these costed millions of dollars. Despite this, in 2017 the country needed N$ 1.2 billion to eliminate malaria to zero cases and the government was able to commit 65% of the funding [13]. However, the country could not reach those targets. Malaria remains a major public health challenge in Namibia mostly in Kavango West and East, Ohangwena and Zambezi region (Fig. 7), despite the fact that it is avoidable and treatable. The four regions in Namibia collectively accounted 96% of the 68 110 and 38 205 malaria cases that were recorded by positive Rapid Diagnosis Tests (RDT) in 2017 and 2018, respectively, were Kavango was topping with 81i% followed by Zambezi with 10% then Ohangwena with 5% [14]. Similarly, the same regions reported the greatest number of total reported cases in 2019, and 2020. We are living in the world of pandemics, and more pandemics are still to come. Hence, fighting other diseases it does not mean we have to neglect the fight of malaria. The malaria confirmed cases by RDT datasets from some years back to date are documented but, to the best of our knowledge, no in-depth studies on spatial and temporal modelling of malaria incidence have been done using 2014 malaria confirmed cases and climatic merged dataset after the 2009 research done by [11]. For example, considering a bigger sample of population at risk of malaria at constituency level in Namibia by looking into results and gaps from the previous researches [11, 15–17].

Disease mapping research has become a common application method used to understand the geographical distribution of a disease, which is typically analysed in the formulation of a Bayesian hierarchical models. Some researchers, [18–22] have employed different approaches and tools such as global and local Moran I statistics, Point pattern analysis, SaTScan Techique, Getis Ordi (Gi*) spatial statistics, and Bayesian hierarchical approach using the Markov Chain Monte Carlo (MCMC) method when they conducted similar studies in other countries and most of these researches found climatic variables to be mostly associated with malaria from spatial and temporal perspectives in their study area.

There were limited number of studies conducted on malaria in Namibia [11, 15–17]. However, none of these studies have considered data of more than 2 years or a larger sample size of the population at risk, and there are no current in depths studies on spatial-temporal using some the latest malaria data since 2016. Therefore, this paper intended fitting a spatial-temporal regression model to profile spatial variation in malaria risk, and investigate possible associations between disease risk and environmental factors at the constituencies level. The 2018–2020 malaria data of all confirmed malaria cases of the current highly malaria known northern regions of Namibia incorporating climatic variables thought to influence malaria distribution from previous studies as well as structured and unstructured random effects was then considered for this study.

## Methods

The analysis in this study was done using the routine surveillance malaria case detection secondary data of people living in high malaria risk Northern regions (Kavango E and W, Zambezi and Ohangwena region) who have visited any of the health facility from 2018 to 2020 reporting fever/suspected to have malaria and tested for malaria using RDT. All recorded malaria positive cases in the 3 years: 34 952 for 2018, 2 990 for 2019, and 10 678 for 2020 from these four regions was then considered.

In this study, several datasets were combined and merged. This includes 2018–2020 malaria weekly surveillance recorded cases (aggregated) dataset obtained from the Ministry of Health and Social Services (MoHSS), 2018–2020 climatic dataset obtained from Southern African Science Service Centre for Climate Change and Adaptive Land Management (SASSCAL), and population dataset for 2018–2020 projected using population estimates of Namibia shape files obtained from Namibia Statistics Agency (NSA).

Malaria is recognized to show regional as well as temporal variation in its distribution, and the target toward eliminating malaria in Namibia can only be easily achieved through conducting in depth researches (spatio-temporal) frequently and the inclusion of environmental covariates improves the model estimates [11, 15, 23]. The function of local factors impacting malaria variability in space and time is better understood in the context of native meteorological conditions [24]. Previously, the Bayesian Conditional Autoregressive (CAR) model was employed to represent geographic heterogeneity in the separate research areas in terms of illness risk. The Bayesian approach provides samples of the entire posterior distribution of incidence rates or relative rates for each area by providing more information than a single point estimate where all parameters are allocated to cope with their likely volatility prior to distribution and this can be achieved through Markov Chain Monte Carlo (MCMC) or Integrated Nested Laplace approximation (INLA). Thus, we fitted a common hierarchical Bayesian CAR model (Besag, York and Mollie’s model) the best model to model the spatio-temporal effect when working with area/lattice data on the 3 years’ combined annual mean (aggregated) data. These data assume different spatial structures to estimate spatial variation in malaria risk and investigate possible associations between disease risk as well as environmental factors at the constituency level, second level administrative unit (see Fig. 8), in Namibia’s highly malaria-infected northern regions, by including fixed effects along with their random effects.

After data cleaning, a neighbourhood structure at the constituency level was created in R software, along with an adjacency matrix and a weight matrix through queens contiguity, but one could also consider comparing different neighbourhood matrix structure e.g., a queen with rook neighbourhood structure. Several measures of spatial correlation were performed, this includes global and local measures of spatial autocorrelation to detect global spatial autocorrelation as well identify constituencies that are spatial clustering. Moran’s I statistics is defined as:

$$I=\frac{n}{S_0}\frac{\sum _{i}\sum _{j}{w_{ij}\left( x_i-\bar{X}\right) \left( x_j-\bar{X}\right) }}{\sum _{i}\left( x_i-\bar{X}\right) ^2},$$

where $S_0$ is the sum of the elements of the weight matrix: $S_0 =\sum _{i}\sum _{j} w_{ij}, w_{ij}$ are the weight matrix entries, and $\bar{X}$ is the mean of the *x* variable. Positive spatial autocorrelation is indicated by Moran’s I coefficient greater than $-1/\left( n-1\right) ,$ whereas negative spatial autocorrelation is indicated by Moran’s I coefficient less than $-1/\left( n-1\right)$. This means that Moran’s $I = 1$ value close to 0 indicates perfect positive spatial autocorrelation, whereas Moran’s I value close to 0 indicates no spatial autocorrelation. Local Moran’s I statistics is defined as:

$$I_{i}\left( d\right) = \left( x_{i}-\bar{x}\right) \sum _{j=1}^{n}{w_{ij}\left( d\right) }\left( x_j-\bar{x}\right) , j\not = i,$$

where $x_i$ and $x_j$ denote the number of count cases at constituency *i* and *j*, respectively, and $w_{ij}$ denotes the spatial weight matrix based on the defined distance lags between constituency *i* and *j* (most of the time lags are in kilometres). If the distance between constituency *i* and constituency *j* is less than *d*, $w_{ij}\left( d\right) =1$; otherwise, $w_{ij}\left( d\right) =0$.

Malaria incidence rate per 1000 population ($({{IR}}_{it} )$ = [number of cases $(y_{it})$ / population at risk $( {P}_{it})$] $\times$ 1000), relative risk (*RR*), and expected cases $({E}_{it})$ of each constituency were computed. Let $y_{it}$ be the count of observed malaria case in each constituency $i (i = 1,2,3,...,34)$ and year $t (t = 1,2,$ and 3), the expected cases of malaria $({E}_{it} )$ can be estimated as the provincial overall mean rate $({mean rate }_t)$ $\times$ constituency population $({pop}_{it})$ and RR $({\theta }_{it} )$ can be computed as $\frac{y_{it}}{{E}_{it}}$. Then, $y_{it}$ could be treated as one realization of poison random variables with means $\mu _{it}$, i.e., $y_{it\ }|{E}_{it}{{,\theta }}_{it} \sim Poisson \left( {E}_{it}{\theta }_{it}\right)$, where $\mu _{it} = {E}_{it} {\times {\theta }}_{it}$ is a function of the effects of k covariates $(x_{kit})$ as well as spatial and temporal random effects. Then we contracted four Bayesian models using climatic variables.

The data failed the premise of equidispersion assumption, as a result, Negative binomial regression a variant of Poisson regression that lowers the Poisson model’s restrictive constraint that variance equals mean was utilised [25–27]. Negative Binomial regression, a special case of Poisson-gamma mixture, assesses the significance of variability in the incidence ratio by modeling Poisson heterogeneity with a gamma distribution and log link function. In this study, the number of observations $(y_{it})$ was assumed to follow a Poisson distribution in the negative binomial model, while the mean $(\mu _{it})$ follows a Gamma distribution [28–31].

The negative binomial distribution is denoted as follows:

1 $$p\left( y \right) = \,\,p\left( {Y = y} \right) = \,\,\frac{{\Gamma \left( {y + \alpha ^{{ - 1}} } \right)}}{{\Gamma \left( {y + 1} \right)\Gamma \left( {\alpha ^{{ - 1}} } \right)}}\left( {\frac{1}{{1 + \alpha \mu }}} \right)^{{\alpha ^{{ - 1}} }} \left( {\frac{{\alpha \mu }}{{1 + \alpha \mu }}} \right)^{y} ,{\text{ }}$$

where $\mu >0$ is the mean incidence rate of *Y* per unit of exposure (e.g., population size area, distance, or time) and $\alpha >0$ is the heterogeneity parameter.

I Spatial model

To provide more information on spatial effects on the data, it is preferable to estimate disease risk by using models that enable to borrow information from neighbouring areas, and incorporate covariates information resulting in the smoothing or shrinking of extreme values based on small sample sizes [32]. The negative binomial model is then expanded to negative binomial Besag-York-Mollie (BYM) spatial model:

2 $$\begin{aligned} \ln (E(y_{{it}} |E_{{it}} ,\theta _{{it}} )) & = \ln (\mu _{i} ) \\ & = \beta _{0} + \beta _{1} x_{{it1}} + \beta _{2} x_{{it2}} + \beta _{3} x_{{it3}} + \cdots \\ & + ~\beta _{k} x_{{itk}} + \sigma \epsilon_{{it}} + off{\mkern 1mu} set(E_{{it}} ) + u_{i} + v_{i} , \\ \end{aligned}$$

where $\mu _{i }$ is the expected annual malaria mean rate, $\beta _0$ is an intercept that explain overall malaria mean rate, $\beta _1, \beta _2,$ ...$, \beta _k$ are estimated parameters (regression coefficients) and $x_{i1 }, x_{i2 }, x_{i3 }\ldots x_{ik }$ are the explanatory/independent climatic variables with corresponding estimated parameters $\beta _k$, $E_{it}$ is the added offset, and $\sigma \epsilon _{it}$ is the disturbance model error that is independent of all covariates, where $exp (\epsilon _{it})$ is assumed to have a gamma distribution with a mean equal to 1 and a smaller variance. The added $u_i$ is the spatial structured random effects while $v_i$ is the spatially unstructured random effects that account for spatial dependence.

Model parameters $\alpha$ and $\beta$ were estimated using maximum likelihood. Let $y_{i}$, $i = 1,2,3,...,n$ be a random variable with probability density function $f(y_{i}|\theta )$, where $\varvec{\theta } = (\theta _{1},\theta _{2},...,\theta _{p}$) is a *p* is a vector of *p* parameters, then the likelihood function is denoted as follows:

$$L \left( \alpha ,\beta \right) = \prod _{i=1}^{n} p\left( y _{i}\right) =\prod _{i=1}^{n}\frac{\Gamma \left( y _{i}+\alpha ^{-1}\right) }{\Gamma \left( y +1\right) \Gamma \left( \alpha^{-1}\right) } \left( \frac{1}{1+\alpha e^{x_{i}\beta }}\right) ^{\alpha ^{-1}}\left( \frac{\alpha e^{x_{i}\beta }}{1+\alpha e^{x_{i}\beta }}\right) ^{y_{i}},$$

The value of $\alpha$ and $\beta$ that maximize $ln L\left( \alpha , \beta \right)$ is the maximum likelihood estimate.

The expectation is $E(y_{i}) = \mu _{i} = E_{i}\theta _{i}$, where $E_{i}$ is the expected rate for the *i* area and $\theta _{i}$ is the relative risk for the $i^{th}$ area.

In (Eq. 2), $u_i$ is assigned a CAR distribution which smoothes the data according to a certain neighbourhood structure that specifies that the two areas are neighbours if they share common boundary: $u_i|u_{-i} \sim N\left( {\bar{\mu }}_{\delta i}, \frac{{\sigma _u}^2}{{n}_{\delta i}}\right)$, where ${\bar{\mu }}_{\delta i} = {n_\delta i}^{-1}\sum _{j \in \delta _{i}} \mu _{j}$, $\delta _{i}$ and $n_{\delta i}$ represent, respectively, the set of neighbours and the number of neighbours for a specific constituency *i*, while $v_i$ is modelled through a Gaussian process as independent and identically distributed normal variables: $v_i \sim N\left( 0, {\sigma _u}^2\right)$ to accommodate extra heterogeneity in the counts due to unobserved risk factors. Priors for the spatial random effects were set to follow log gamma distribution with mean = 0, precision = 0.001 (since it’s a Negative Binomial model), while the default prior assigned to the associated coefficients (and the intercept) was a Gaussian distribution.

II Spatio-temporal model

The Negative Binomial Besag-York-Mollie (BYM) spatial model (Eq. 2) was extended to allow for a temporal component:

3 $$\begin{aligned} \ln (E(y_{{it}} |E_{{it}} ,\theta _{{it}} )) = \ln (\mu _{{it}} ) & = \\ & \beta _{0} + \beta _{1} x_{{it1}} + \beta _{2} x_{{it2}} + \beta _{3} x_{{it3}} + \ldots + \beta _{k} x_{{itk}} + \sigma \in _{{it}} \\ & + ~offset(E_{{it}} ) + u_{i} + v_{i} + \left( {\beta + \delta _{i} } \right) \times t, \\ \end{aligned}$$

where $( \beta + \delta _i)\times t$ is the added main linear trend. In this equation, $\beta$ represent the global time effect and $\delta _i$ is the differential trend that identifies the interaction between time and space.

III Full Spatio-temporal model (linear predictor of non-parametric trend: model with interaction)

This is the model with interactions, for example, nonspatially or temporally structured interaction and temporally structured interaction. The nonspatially or temporally structured interaction assumes that the unstructured spatial effects ${(v}_i)$ is interacting with the unstructured temporal effect ${(\phi }_t)$ and the structure matrix is denoted as follows:

$$\begin{aligned} R_\delta \ =R_u\ \bigotimes \ \ R_\phi \ =\ I\ \bigotimes \ I\ =\ I. \end{aligned}$$

Since we assumed not spatial neither temporal structure on this interaction, then:

$$\begin{aligned} \delta _{it}\sim N\left( 0\mathrm {,\ \ }\frac{1}{\tau _\delta }\right) . \end{aligned}$$

This means that the unstructured interaction can be viewed as unobserved independent factors for each constituency and year combination, resulting in no structure [33]. However, if the model includes spatial and temporal main effects, this interaction effect simply suggests independence in deviations from them [33]. Due to the main effects, contributions to malaria risk in neighbouring constituencies or in subsequent years (e.g., 2018, 2019, and 2020) can still be highly connected. As a result, this is a global space-time heterogeneity effect that is typically modelled as white noise.

The temporally structured interaction assumes that the unstructured spatial effect ${(v}_i)$ is interacting with the structured temporal effect $(\gamma _t)$ and the structure matrix is denoted as follows:

$$\begin{aligned} R_\delta \ =R_v\ \bigotimes \ \ R_\gamma \, \end{aligned}$$

where $R_v = I$ and $R_\gamma$ is the neighbouring structure defined through random walk of order two *(RW2)*. This results in the assumption that for the $i^{th}$ constituency, the parameter vector $\left\{ {\delta _{1i},\ldots , \delta _{ni}}\right\}$ has a time-dependent autoregressive structure component which does not depend from the ones of the other constituencies [34].

In this interaction, each zone (constituency) has its own structure that is distinct/independent from nearby constituencies, and the evolution structure for each constituency can take on as many forms as the temporal main impact itself. However, this does not imply that each constituency evolves independently of the others, as they may have a similar temporal main effect. Independence has little effect on deviations from the global trend [33].

Full spatio-temporal model (model with interaction) is then denoted as follows:

4 $$\begin{aligned} ln ( E(y_{it}|\theta _{it}))= \,{} ln (\mu _{it} ) \nonumber \\= \,{} \beta _0+\beta _1x_{1it }+\beta _2x_{2it}+\beta _3x_{3it}+\ldots +\beta _kx_{kit } +\sigma \epsilon_{it} \nonumber \\{} {} + ~ u_{i} +v_{i}+\gamma _{t} + \phi _{t}+\delta _{it}, \end{aligned}$$

where ${\gamma }_t$ represent the structured temporal effect modelled dynamically using RW of order 2 to allow for extra heterogeneity in the counts due to unobserved (and spatially unstructured) defined as: $\gamma _{t } |\gamma _{t- 1}, \gamma _{t- 2 }\sim N ( 2\gamma _{t- 1} +\gamma _{t- 2 }, \sigma ^2)$, and was assumed to be an autoregressive process while $\phi _t$ represent the unstructured temporal effect and was specified by means of Gaussian exchangeable prior, defined as: $\phi _t\sim N\left( 0\mathrm {,\ \ }\frac{1}{\tau _{\phi _t}}\right)$. To allow for interaction between space and time, which explain differences in the time trend of malaria risk for different constituencies. The parameter $\delta _{it}$ follow a Gaussian distribution with a precision matrix given by $\tau _\delta R_\delta$ where $\tau _\delta$ is unknown scalar, while $R_\delta$ is the structure matrix, identifying the type of temporal and/or spatial dependence between the elements of $\tau _\delta R_\delta$ can be factorized as the Kronecker product of the structure matrix of corresponding main effects which interact.

## Results

Descriptive analysis were performed and model parameters were estimated using Integrated Nested Laplace approximation (INLA) in R software since Bayesian estimation using INLA takes less time as compared to Markov Chain Monte Carlo Methods (MCMC). The best model was selected using the deviance information criterion (DIC) given by: $DIC = D + 2p$, where D is the deviance that is evaluated at the posterior mean and p is the effective number of parameters where model with smallest DIC value was considered as the best model that fit the data.

### Descriptive statistics

**Fig. 1:**
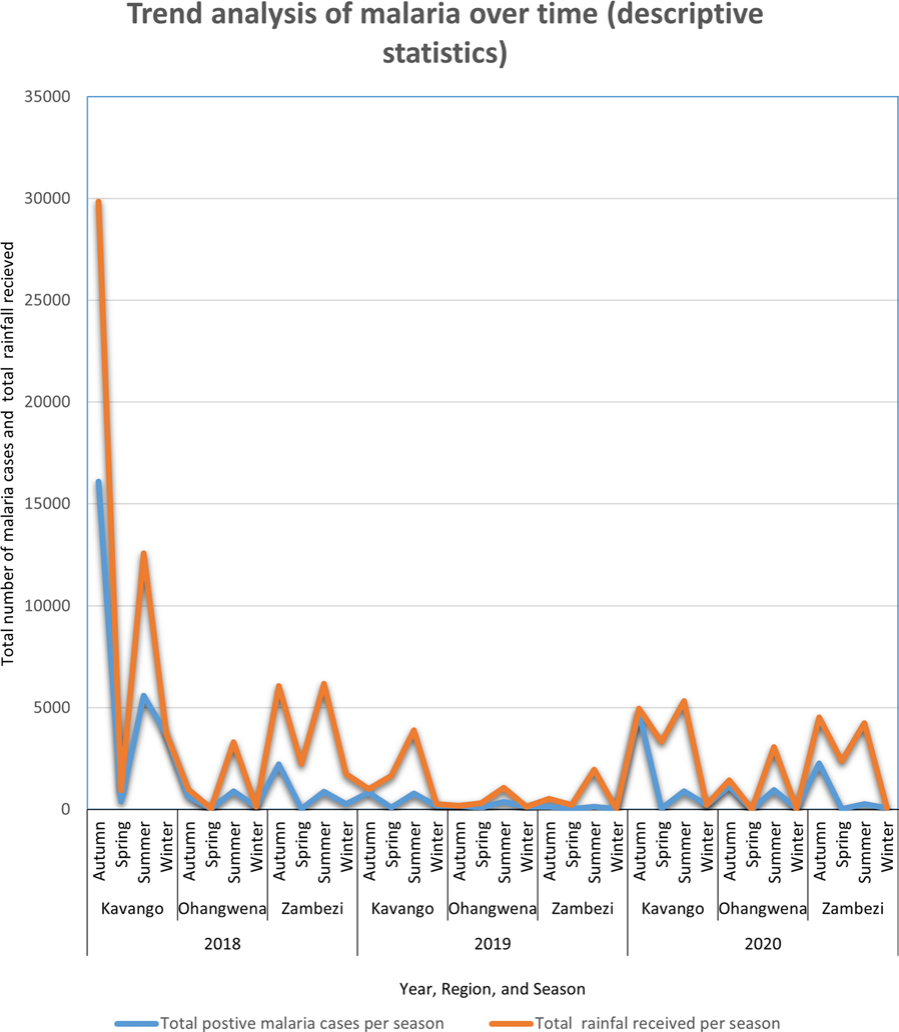
Trend analysis of malaria (2018–2020)

It is highly known that there is correlation between malaria and rainfall in most countries. The observed number of malaria from 2018–2020 in Namibia was also found to be directly proportional to the amount of rainfall received, the high the rainfall the more cases of malaria were observed (see Fig. 1).

**Fig. 2:**
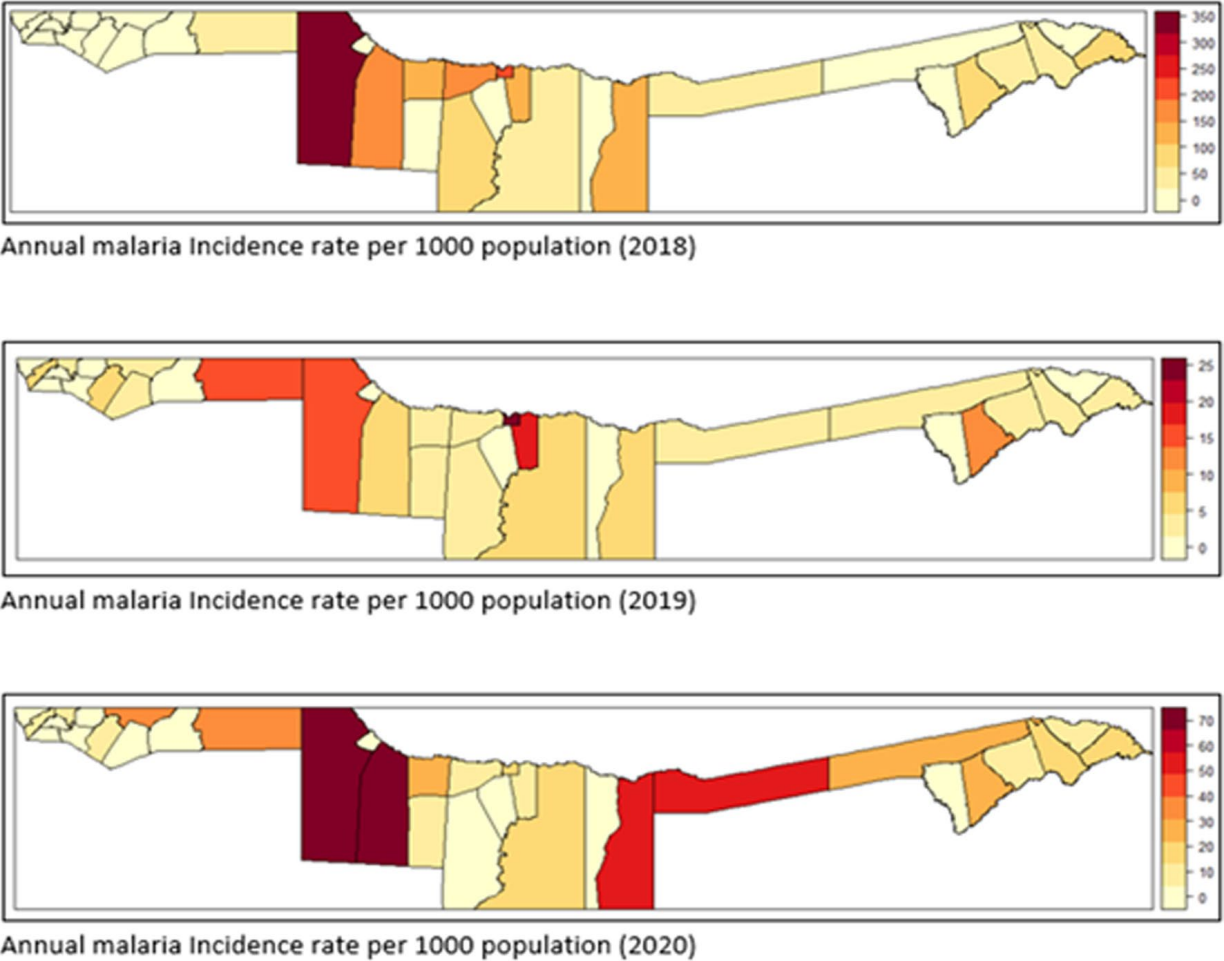
Malaria Incidence rate per 1000 population at constituency level (descriptive analysis)

**Fig. 3:**
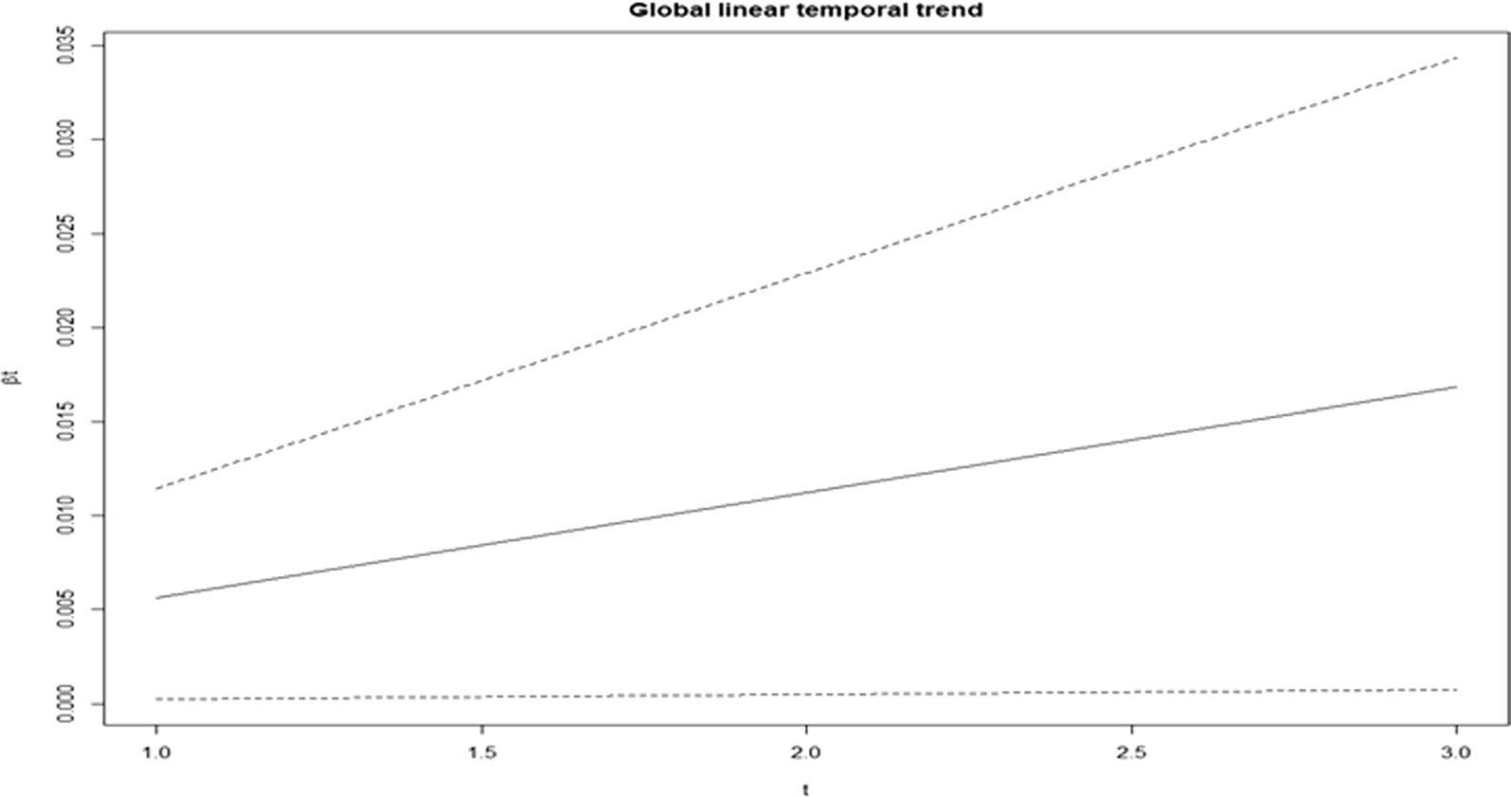
Global linear temporal trend for malaria (spatio temporal model with added covariates)

**Fig. 4:**
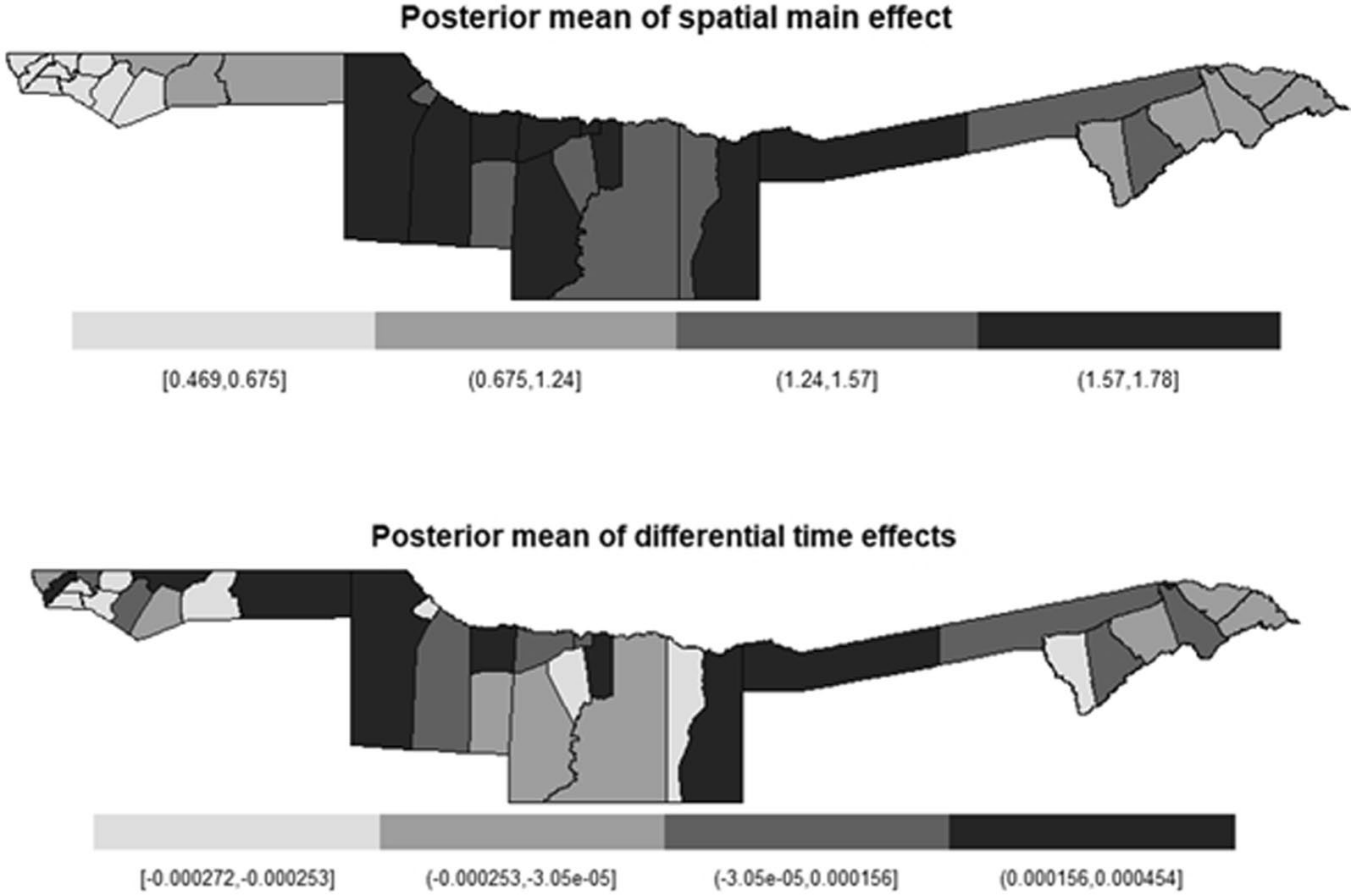
Spatial main effect and differential temporal maps of the spatiotemporal

**Fig. 5:**
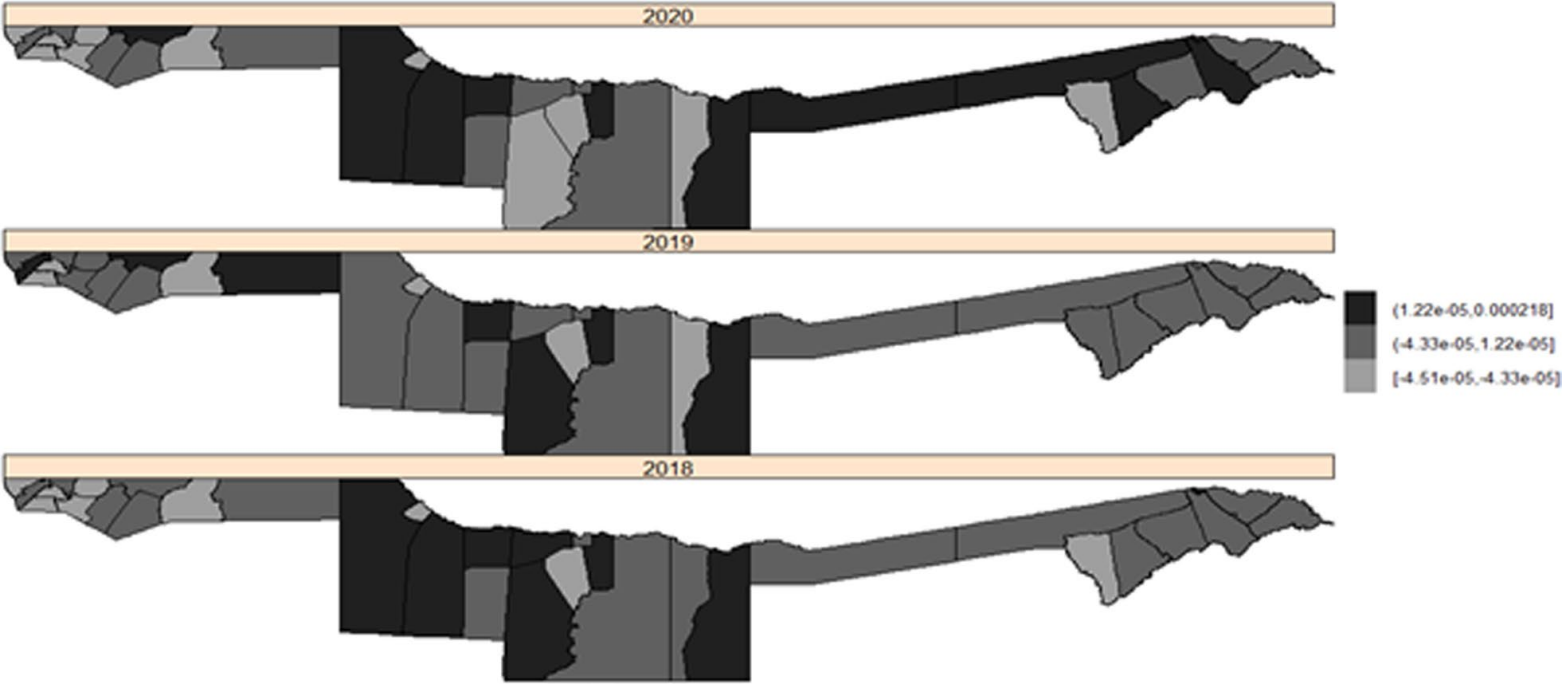
The maps for the posterior mean relative risk of unstructured interaction (spatio-temporal model)

**Fig. 6:**
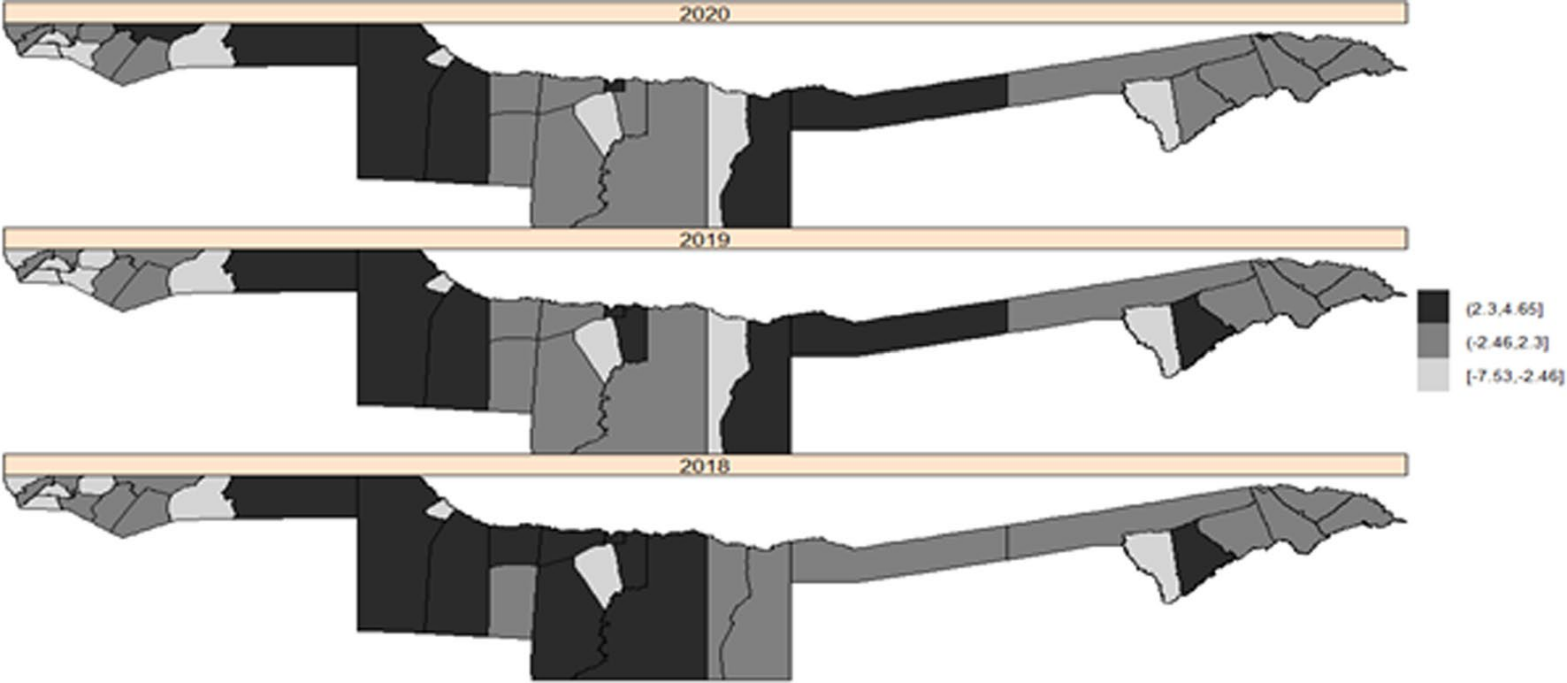
The maps for the posterior mean relative risk of temporally structured interaction (spatio-temporal model)

**Fig. 7:**
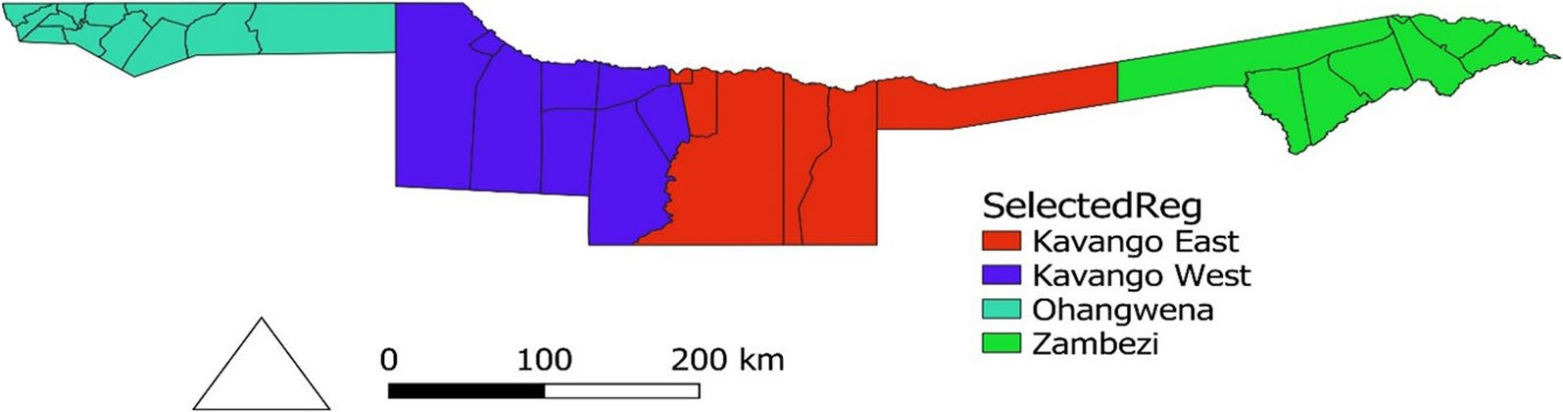
Map of the 4 regions (Kavango West and East, Zambezi and Ohangwena)

**Fig. 8:**
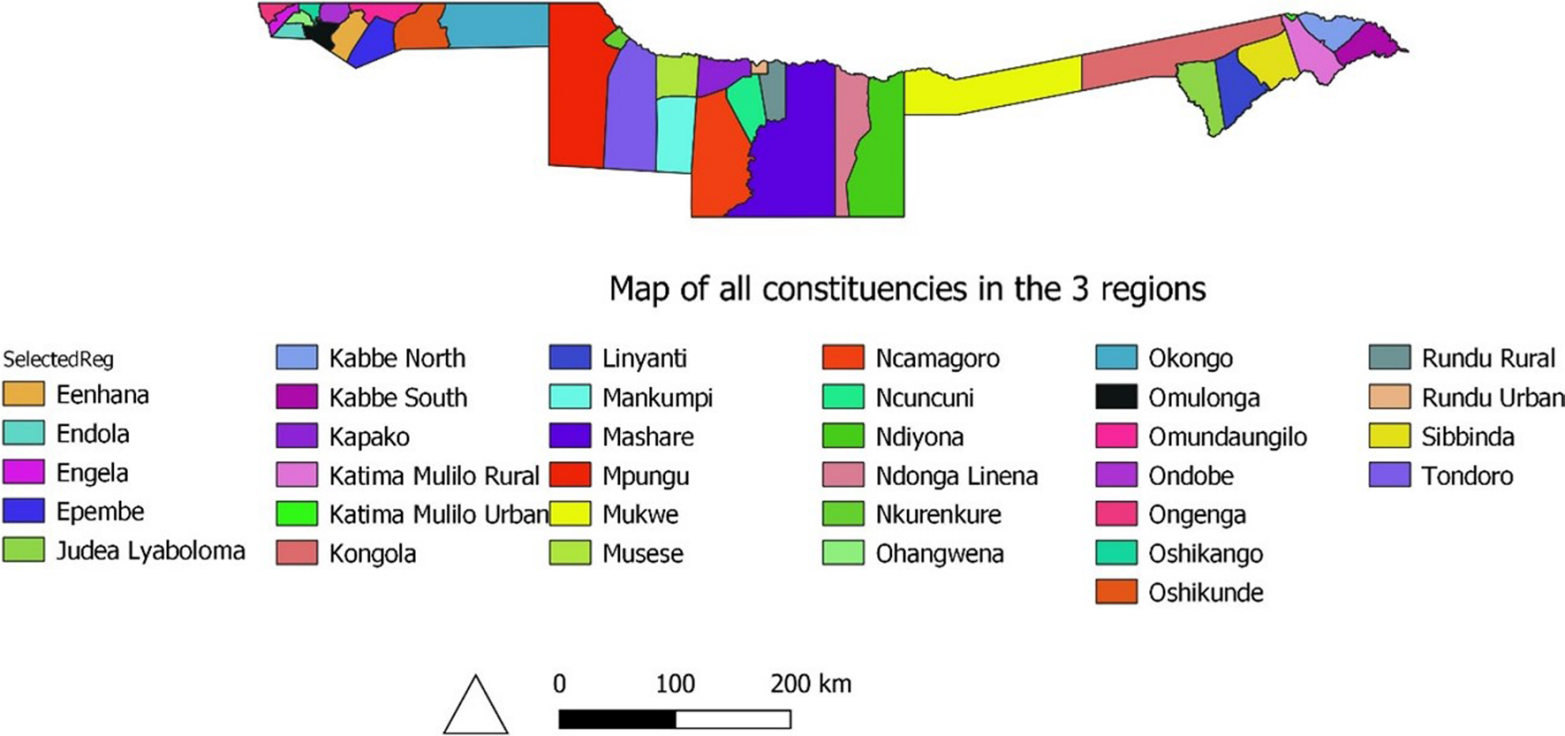
Map of all constituencies in the 4 regions

Results presented in Table 1 show that the three-year average incidence rate (2018–2020) was found to be 6 cases per 1000 population, with the incidence rate being very high in 2018, 12 cases per 1000 population, and decreasing to 1 case in 2019, but unfortunately increasing again in 2020 from 1 case to 4 cases per 1000 population. Mpungu constituency had the highest annual average malaria incidence, 137 cases per 1000 people, for a 3 year period (2018–2020), followed by Tondoro, Rundu urban, and Rundu rural, with 79 and 78 cases per 1000 population, respectively, highlighted in dark red and red (Fig. 2).

**Table 1:** Summary of computed malaria incidence per year

Year	Estimated population	Number of cases	Mean rate	IR/1000 population (Namibia)	IR/1000 population in 4 regions
2018 - 2020	2494579	14881	0.00597	6	23
2018	2448301	31619	0.01291	12	49
2019	2494530	2990	0.00120	1	5
2020	2540905	10035	0.00395	4	15

*IR* Incidence rate per 1000 population

**Table 2:** log scale parameter for the best negative binomial BYM spatial model

Parameter	Spatial model with covariates				
			Quantiles		
	P mean	SD	25	50	75
Intercept	−53.3	11.8	−78.47	−52.61	−31.98
HPD	0.000	0.001	−0.002	0.000	0.003
ST (avg)	0.011	0.014	−0.017	0.012	0.038
T (avg)	0.982	0.491	0.040	0.972	1.978
T (min.)	0.053	0.187	−0.299	0.047	0.438
T (max.)	0.840	0.198	0.524	0.812	1.296
RF	0.058	0.026	0.011	0.057	0.113
WS (avg)	1.068	0.253	0.658	1.037	1.645
H	−0.089	0.036	−0.162	−0.088	−0.020
LW (avg)	−0.015	0.006	−0.028	−0.015	−0.004
PoNB	0.782	0.166	0.498	0.768	1.15
$u_{i}$	828.0	753.8	61.3	614.6	2857.2
$v_{i}$	931.5	859.6	76.0	688.2	3182.4

*P mean (Posterior mean )*

*SD (standard deviation )*

*PoNB (Parameter of Negative binomial)*

$u_{i}$ *(structured random effect)*

$v_{i}$ *(unstructured random effect)*

**Table 3:** log scale parameter for the Bayesian negative binomial (BYM) unstructured interactive spatio-temporal model

Full Spatio-temporal model					
			Quantiles		
Parameters	P mean	SD	25	50	75
Intercept	0.436	0.661	−0.826	0.423	1.77
T (avg)	0.000	0.000	0.000	0.000	0.000
T (max.)	0.006	0.006	0.005	0.005	0.017
RF	0.006	0.003	0.000	0.005	0.011
WS (avg)	−0.003	0.003	−0.009	−0.003	0.004
H	0.002	0.004	−0.006	0.002	0.009
LW (avg)	−0.001	0.001	−0.002	−0.001	0.001

**Table 4:** Negative binomial regression non-spatial model (Individual malaria data)

Results of the individual dataset			
Parameters	$\hat{\beta }$	P-value	95% CI
Intercept	0.032	<0.001	−3.577, −3.298
Gender			
Male	1.089	0.001	0.035, 0.136
Female (Ref)	1.000		
Age group			
5 to 19	1.236	<0.001	0.136, 0.289
20 to 39	1.112	0.018	0.018, 0.195
40 to 59	1.027	0.636	−0.083, 0.135
60 and above	0.881	0.056	−0.258, 0.002
0 < 5 years (REF)	1.000		
Place of residence			
Village	1.030	0.307	−0.027, 0.088
Town (Ref)	1.000		
Type of health facility			
Health centre	1.088	0.029	0.008, 0.160
Hospital	1.130	<0.001	0.063, 0.182
Clinic (Ref)	1.000		
Occupation			
Mosquito-infested employees	0.705	<0.001	−0.476, -0.225
Professionals	0.689	<0.001	−0.571, -0.182
Small business	0.898	0.004	−0.181, -0.035
Unemployed	0.894	0.001	−0.181, -0.043
Youth (REF)	1.000		
Slept under mosquito bed net in the last 3 night			
Yes	1.404	<0.001	0.266, 0.414
No (Ref)	1.000		
Home sprayed in past 12 months			
Yes	0.990	0.718	−0.062, 0.043
No (Ref)	1.000		
District			
Eenhana	1.132	0.115	−0.033, 0.276
Engela	0.847	0.087	−0.361, 0.020
Katima - Mulilo	1.010	0.931	−0.215, 0.223
Nankudu	2.083	<0.001	0.646, 0.821
Nyangana	2.055	<0.001	0.629, 0.811
Okongo	1.106	0.356	−0.120, 0.309
Rundu	1.383	<0.001	0.246, 0.403
Andara (Ref)	1.000		
Season			
Spring	0.808	0.046	−0.428, -0.010
Summer	0.998	0.946	−0.058, 0.054
Winter	0.829	<0.001	−0.269, -0.107
Autumn (Ref)	1.000		

Using the spatial approach, several measures of spatial correlation were performed. The Global Moran’s I statistics value was found to be positive: 0.1863 (p - value = 0.0429) with a variance of 0.0159. This indicates that there was spatial autocorrelation in the data at the constituency level, indicating that malaria was clustered in these 3 Northern regions of Namibia. Comparing these results to previous similar studies conducted in Namibia, the clustering area were now quite very small in some Northern parts compared to result found by [11, 17] and this can help the MoHSS and NVDCP team to effortlessly convey out intervention using the available partial resources in these small areas.

### Model results

After fitting different models, for both analysis (spatial and spatial- temporal), the models with added climatic factors revealed the smallest DIC values. The results of the best spatial and temporal model (models with smallest DIC values for both analysis) using malaria aggregated data (2018–2020) are presented in Tables 1 and 2.

From the spatial Negative Binomial BYM model resuts, annual monthly average temperature (mean), annual monthly maximum temperature (mean), annual monthly total rainfall (mean), and annual monthly average wind speed (mean) all had a significant positive effect on annual mean malaria incidence, whereas annual monthly average humidity (mean) and annual average leaf wetness (mean) had a significant negative effect (Table 2). However, annual monthly soil temperature (mean) and annual monthly minimum temperature (mean) were both found to be positively related to malaria annual mean incidence rate, but this was not significant, and human population density was found to have no effect on malaria incidence rate in North Namibia (Table 2).

From Table 3, only total rainfall and maximum temperature were found to have a significant effect on malaria through space and time. For every one $^\circ C$ and mm increase in annual temperature as well as rainfall in a certain constituency, it will increase the annual mean cases of malaria by 0.6%. The remaining variables did not show a significant relationship with malaria cases from spatial and temporal perspectives at 95% confidence intervals.

The plot of the posterior mean of the main time effect in years (Fig. 3) clearly indicates a slight increase in global trend as time passes, for example, a high estimate of malaria was observed from 2018 to 2020. However, $\xi = u_i+ v_i$, the posterior mean of the spatial (structured and unstructured) obtained using a random walk of order 2 effects revealed a greater spatial effect in constituencies located on the outskirts of Kavango East and Kavango West with relative risk ranged from 1.57 to 1.78 (Fig. 4). Regrettably, the two regions on the east and west sides of the Kavango, two in the middle of the Kavango, and two in the Ohangwena region showed a higher differential trend than the average (Fig. 4).

In 2018 and 2020, a pattern of positive significant unstructured random effects (spatial and temporal) was observed, primarily in the west of Kavango and constituencies bordering Kavango East with Zambezi region. Furthermore, an increase in the number of constituencies having turned black (high risk/positively significant) was observed between 2018 and 2020. However, in some of the Kavango and Ohangwena constituencies, a negative significant unstructured random effect was detected (negative posterior mean in light grey), see Fig. 5. Also, a pattern of temporal structured random effects was observed in both years and there were no changes in the constituencies at high malaria risk in 2019 and 2020 (Fig. 6).

## Discussion

The four regions Kavango East and West, Ohangwena, and Zambezi reported a total of 44 644 malaria cases over a three-year period, with 31 619 instances reported in 2018, 2 990 in 2019, and 10 035 in 2020. This accounted for an average of approximately 90% of all reported malaria cases in Namibia throughout the last 3 years (2018–2020). Namibia was indeed expected to have 0 cases per 1000 population in 2020. A 92% incidence rate decrease was achieved in 2019 as the incidence decreased from 12 cases per 1000 population in 2018 to 1 case in 2019, but unfortunately, the incidence rate has increased again from 1 case in 2019 to 4 cases per 1000 population in 2020 (Table 1) with a high malaria transmission still in Kavango West and East, Ohangwena and Zambezi constituencies.

Malaria risk predicted from spatio-temporal models (Fig. 5) observed an increase in the number of constituencies having turned black (high malaria risk) in the east of Kavango in 2020 compared to 2018 and 2019. Results from the spatio-temporal unstructured interactive model presented in Fig. 6 has predicted high malaria risk in some of the Zambezi constituencies eg., Kongola, Katima-mulio urban, Katima-mulio rural, and Sibbinda in 2020 that could not be detected by the incidence map in Fig. 2 because of high rainfall and temperature together with some unstructured spatial and random interactions in those constituencies that were added to the model to improve the fit. The spatio-temporal structured interactive model (Fig. 6) also predicted high malaria risk in some of the Kavango and Zambezi constituencies including Ongenga, Engela, Ondobe, Omulonga, and Epembe constituency in Ohangwena region in 2019 and 2020 although unstructured interactive risk map (Fig. 5) explains the malaria risk better than the temporal structured interactive risk map (Fig. 6).

Moreover, similar studies on malaria spatial modelling conducted earlier in African and Asian countries e.g [18, 20–22] have found malaria distribution to have high peak during rainy season. Results of this study also evidenced that rainfall has an effect on the distribution of malaria through space and time with high malaria cases being reported during Summer and Autumn (December to May) as compared to other season and the country record high annual rainfall the same period (December to May). Furthermore, the study found spatial and temporal variation of malaria risk to be due to a combination of climatic factors both observed and unobserved where, average annual total rainfall and annual average maximum temperature were found to explain spatial and temporal variation of malaria infection in Namibia from both temporal and spatial perspectives and this was similar to the results obtained by [20] in Malawi. In addition, previous studies conducted before e.g [11] using the 2009 malaria data revealed that high populated areas were more at risk of Malaria. However the model fitted for this study [11] found population density to have no effect on malaria cases and this could be due to different period (2018–2020), different dataset used, the model employed, and variety of variables that were added to improve the model fit (Table 1). The population living in the far East and West of Kavango, and Zambezi region constituencies were predicted to be more at malaria risk as compared to others (Fig. 4, 5, and 6). This confirmed that the occurrence of malaria cases in constituencies might be high closely related to the two ecological factors maximum temperature and amount of rainfall received as those were the constituencies that annually receive high rainfall and annual record high temperature.

The current study aimed at spatial-temporal regression model to characterize geographical variation in malaria risk and evaluate possible connections between disease risk and environmental factors at the constituency level in Northern Namibia highly malaria-endemic areas. Through the whole posterior inference approach, a detailed examination of the uncertainty in the unobserved random factors that also contribute to the volatility of malaria mean rate was done. The procedure was accomplished by adding the observed and unobservable random effects of fascinating into the whole hierarchical Bayesian model. The random effects that were evaluated included structured space-time heterogeneity, which measured the effect of constituencies clustering, unstructured heterogeneity, time trend effect, which represented the three respective periods 2018–2020, interaction between space and time, and the covariates effect of climatic variable, as well as some other possible variables that were found to have a significant effect on malaria in other countries according to literature. R-INLA was used to perform the analysis, which included prior and hyper-prior distribution specifications for the parameter and hyper parameters.

As one of many preventive measures, MoHSS has been using indoor residual house spraying (IRS) for a long time to lower the number of malaria mosquitoes that transmit the disease. Yet, the usage of IRS could harm the environment and result in pesticide resistance [35]. A non-spatial model using individual data considering some of these measures variable was fitted. The variable gender, age group, place of residence, type of health facility, occupation, employment status, sleeping under mosquito bed nets, and district were all found to be significantly associated with malaria incidence. Specifically, The log expected count of males were found to be 1.089 times more likely to be infected than females after controlling other variables at (95% CI, and p- value < 0.001). Unlike in other countries where less than 5 years old Children are more likely to be on malaria risk, the log expected count of Individuals aged 5 to 19 years in Namibia were found to be 1.236 times more likely than individuals aged less than 5 years, and this was significant at 5% level of significance. The rate of testing positive for malaria in a villager was found to be 3% higher than in a town dweller, but there was insufficient evidence to conclude this at 5% level of significance (Table 4). Results also revealed that uses of mosquito nets and practising of residual house spraying does not lower malaria incidence as much (Table 4).

Malaria transmission is unstable, seasonal, characterized by outbreaks and concentrated in 7 endemic northern Namibia regions [35] mostly the four regions considered in this study. Alternative interventions such as the use of biological agents which are more friendly to the environment to treat the mosquito breeding sites were explored as a way to malaria elimination target. A demonstration larviciding project aiming to increase the involvement of the community in malaria mosquito control interventions was recently implemented in five malarious districts selected from 4 regions namely Omusati, Oshikoto, Ohangwena and Kavango East through [35]. Field team implements the adult mosquito collection and larviciding technique in their respective villages and treat the active mosquito breeding sites and continue monitoring them until they are mosquito larvae free [35]. The team is also collecting adult mosquitoes from 20 randomly selected households in the respective five villages to monitor the mosquito population density and to assess the impact of both the larviciding and IRS. Therefore, the MoHSS with the help of the National Vector Borne Disease Control Programme (NVBDCP) and the government should extend this project to the identified new malaria risk constituencies (hotspots) from both analyses of this study. Some full interventions malaria package must also first prioritized to individuals living in the identified malaria hot spots constituencies mostly constituencies with average/high temperature, and low humidity at the appropriate time (January - May “rainy season”) more specifically individuals aged 5 to 19, the youth e.g., children, learners, and students, and individuals that are not employed or working in Small Business (e.g., small market sales, traders, and other manual labourers, specifically males as these are the people that were found to be more exposed to the malaria risk (Table 4).

Moreover, the current study was a non-funded study that only focused on the available malaria secondary data. Hence, in addition to the above recommended interventions, MoHSS with the help of NVBDCP and the government must come through with some funds that will enable researchers to team up or join the larviciding project members and conduct deep survey in those identified hotspot constituencies capturing all primly reverend information on all possible putative sources of malaria transmission that was missed in the current study due to lack of data on key variables such as potential anopheles mosquito habitat, residual transmission foci, exports or imports malaria infections, and many more. These information can be merged to obtain a monthly or weekly spatio-temporal mapping at village/town level every one year or quarterly in order to help MoHSS achieve malaria SDG 3 that aims to have zero malaria cases by 2030.

## Conclusions

The current study discovered that the spatial temporal model with both random and fixed effects best fit the model which demonstrated a strong spatial and temporal heterogeneity distribution of malaria cases (spatial pattern) with high risk in most of the Kavango West and East outskirt constituencies where high malaria peak was discovered to occur during Autumn and Summer (January to May). Annual average rainfall, annual average maximum temperature together with some unobserved random effects were found to be significantly associated with malaria cases distribution through space and time. Furthermore, the findings of the best BYM model’s posterior mean estimations of the parameters revealed that unstructured random effects contributed to most of the malaria variation in Namibia.

The use of a Bayesian approach to estimate the contributions of environmental indicators on the spatial-temporal pattern of pandemic diseases should be encouraged in statistics since it consider sensitivity data errors as a way to guide appropriate actions and better allocation of limited healthcare resources.

Finally, future research should take into results and lessons from the larviciding experiment currently implemented in few villages mostly focusing on discovering and mapping probable anopheles mosquito habitat using geographical and temporal survival analyses while also examining all other suspected causes of malaria transmission. In order to ensure high accuracy of the verified/confirmed cases, future research should also utilize Polymerase Chain Reaction (PCR) test of malaria. Additionally, to ensure malaria data quality with all key variables, high-quality malaria projects and programs should be implemented in these designated malaria hotspot constituencies and MoHSS must engage closely with NVBDCP Officers and other Consultants at various levels.

## Limitation

The aggregated data indicated that the four regions (Kavango West and East, Ohangwena, and Zambezi) account for more than 80% of all malaria cases recorded since 2017 to 2020. According to statistics from the Ministry of Health and Social Services, of the 38 204 cases reported in Namibia in 2018, the four Northern regions contributed 96% of the total cases reported, with the two Kavango West and East leading with 81%, followed by Zambezi with 10%, and Ohangwena with 5%, with the remaining regions accounting for only 4% of the total cases reported. Therefore, this study was restricted to those areas. Also, mosquito breeding sites could not be mapped due to lack of additional key variables in the available data.

## Data Availability

Malaria dataset are available on DHIS2, climatic data are available (SASSCAL Weather Net page http://www.sasscalweathernet.org/index.php?MIsoCode=NA), and Namibia shapefiles can be obtained from Namibia Statistics Agency office.

## Abbreviations

BYM: Besag-York-Mollié model
CAR: Conditional auto-regressive
DIC: Deviance information criterion
DHIS2: District health information software 2
INLA: Integrated Nested Laplace approximation
IRS: Indoor residual house spraying
MCMC: Markov chain Monte Carlo
MIS: Malaria indicator survey
MoHSS: Ministry of health and social services
NVBDCP: National vector borne disease control programme services
NSA: Namibia statistics agency
PCR: Polymerase chain reaction
RDT: Rapid diagnosis test
SASSCAL: Southern African Science cervice centre for climate change and adaptive land management
SDG 3: Sustainable development goal 3
WHO: World Health Organization
